# Trichomoniasis associated with amniotic sac rupture in pregnancy: a case report

**DOI:** 10.1186/s12884-026-08780-4

**Published:** 2026-07-13

**Authors:** Hajar Ziaei Hezarjaribi, Maliheh Ghasemi Tirtashi, Marzieh Asadi

**Affiliations:** 1 Department of Parasitology, Toxoplasmosis Research Center, Communicable Diseases Institute, Mazandaran University of Medical Sciences, P.O. Box: 48175-1665, 18th Km of Khazarabad Road, Sari, Mazandaran Province Iran; 2 Iranian National Registry Center for Lophomoniasis and Toxoplasmosis, Imam Khomeini Hospital, Mazandaran University of Medical Sciences, Sari, Iran; 3 Sexual and Reproductive Health Research Center, Mazandaran University of Medical Sciences, Sari, Iran

**Keywords:** Trichomonas vaginalis, Pregnancy, Sexual transmission, Premature rupture, Amniotic sac, Case report

## Abstract

The aim of this report is to present a rare case of pre‑labour premature rupture of membranes (PPROM) in a patient in the second trimester of pregnancy, in which an association with a lower genital tract infection caused by *Trichomonas vaginalis* was observed. The patient was a 26‑year‑old woman, gravida 2, at 25 weeks of gestation, who presented with clinical complaints of abnormal vaginal discharge and fluid leakage.

Following a definitive diagnosis of trichomoniasis based on stained smear and culture findings, standard treatment with metronidazole was initiated. The treatment was effective, leading to complete resolution of maternal symptoms, full eradication of the infection, and a stable, viable fetal condition.

This case highlights the potential importance of timely identification and management of common sexually transmitted infections during pregnancy, as such infections may be associated with alterations in fetal membrane integrity and adverse pregnancy outcomes, although a causal relationship cannot be inferred from a single observational report.

**Supplementary Information:**

The online version contains supplementary material available at https://doi.org/10.1186/s12884-026-08780-4.

## Introduction

Trichomoniasis, caused by the protozoan parasite *Trichomonas vaginalis*, is one of the most important sexually transmitted infections (STIs) worldwide [1]. *T. vaginalis* exists only as a motile trophozoite (with no cyst stage), replicates by longitudinal binary fission, and is transmitted predominantly through sexual contact, although rare cases of nonsexual transmission via contaminated moist surfaces have also been reported [1, 2]. A notable feature of trichomoniasis is the high rate of asymptomatic infection. Estimates suggest that 50–75% of infected women may be asymptomatic. This asymptomatic nature reinforces the cycle of disease transmission and makes screening and treatment a major challenge [1, 3].

In women, trichomoniasis most commonly presents with a foul‑smelling, frothy vaginal discharge, pruritus, dyspareunia, and dysuria, though it may be asymptomatic; in men, it often manifests as urethritis with urinary discharge or dysuria and can serve as a source of transmission. While in the general population it is typically viewed as a health problem associated with vaginitis or urethritis, when diagnosed during pregnancy its significance increases, given reported associations with adverse pregnancy outcomes and complications [3, 4]. The active presence of *T. vaginalis* in the genital tract of pregnant women is considered a predisposing factor for the onset of labor processes due to the inflammatory mechanisms and local damage it causes [4].

Structural damage and inflammation possibly associated with the secretion of *T. vaginalis* proteases in the vaginal and cervical mucosa may weaken the defense barrier and increase susceptibility to secondary pathogens. This infection may contribute to increased levels of proinflammatory cytokines such as IL‑1 and IL‑6 in the amniotic fluid, which have been associated with mechanisms involved in the onset of labor [5]. This inflammatory cascade increases the risk of preterm labor by up to twofold. Also, the activity of the parasite’s proteolytic enzymes has been associated with an increased risk of premature rupture of membranes (PROM) and, consequently, low birth weight (LBW). Overall, trichomoniasis severely compromises the health of pregnancy by causing local and systemic inflammation [6].

Given the serious risks involved, any optimization of the diagnostic and therapeutic protocol that leads to successful eradication of the parasite would directly impact the reduction of this perinatal mortality and morbidity. The management of sexually transmitted infections during pregnancy has always been complicated by pharmacological constraints due to the need to protect the health of the fetus. Trichomoniasis is no exception. The standard treatment for trichomoniasis in non-pregnant individuals is a single, high-dose dose of metronidazole. Metronidazole is in the US Food and Drug Administration (FDA) category B [1]. However, its use in the first trimester of pregnancy has always been associated with concerns about teratogenicity (the creation of birth defects), especially at the high doses required to treat chronic or resistant infections. Although extensive postnatal studies have ruled out a definitive and strong association between metronidazole and birth defects, clinical caution still prevails. Conventional treatment protocols often recommend that metronidazole treatment be delayed beyond the 12th week if possible or that lower doses and longer durations be used [1, 7]. Given the clinical importance of trichomoniasis during pregnancy, this case report presents a pregnant woman with confirmed *T. vaginalis* infection and aims to contribute to increased awareness, shared clinical experience, and strengthened knowledge regarding the management of this infection in vulnerable maternal populations. What makes this case particularly notable is the occurrence of premature rupture of membranes (PROM) at 25 weeks’ gestation in probable association with *T. vaginalis*, despite the absence of other commonly recognized risk factors. By documenting this case, we highlight the potential role of *T. vaginalis* as an associated factor in PROM and underscore the importance of timely screening and effective treatment during pregnancy to help reduce the risk of related complications.

## Case presentation

This case report is derived from our original study entitled “Case Report: Diagnosis and Management of Trichomoniasis in a High-Risk Pregnancy.” The study was approved by the Ethics Committee of Mazandaran University of Medical Sciences (ethical code: IR.MAZUMS.REC.1404.517) and was conducted in accordance with the ethical principles of the Declaration of Helsinki. Written informed consent was obtained from the patient for publication of clinical details.

A 26-year-old female patient with a history of one previous delivery presented at the 25th week of her current pregnancy with the chief complaint of vaginal discharge. According to the patient’s medical history, she had no documented history of Pap smear, sexually transmitted infection (STI) screening, or vaginal examination prior to or during the early stages of the current pregnancy. At presentation, she also reported burning and itching, increased vaginal discharge, and urinary urgency. To reduce the impact of potential confounding factors, common risk factors associated with premature rupture of membranes (PROM) were systematically assessed. No other known risk factors for premature rupture of membranes were identified. A summary of the risk factors assessed, the associated assessment methods, and the results are presented in Table 1.

**Table 1:** Assessment of potential confounding factors for premature rupture of membranes

Risk factor	Assessment method	Result
Bacterial vaginosis	Vaginal culture or microscopy	Negative
Cervical insufficiency	Clinical history and ultrasound	Not present
Smoking	Self-report	No
Prior preterm birth	Obstetric history	None

Clinical examination with a speculum revealed thick, watery, foamy, and yellowish-gray discharge, and the vaginal mucosa was inflamed. The POOLING TEST was positive, but due to the abundant vaginal discharge, other tests were also used to confirm premature rupture of the amniotic sac, including the Fern Test, AmniSure Test, and Amniotic Fluid PAD TEST, all of which confirmed premature rupture of the amniotic sac.

**Fig. 1:**
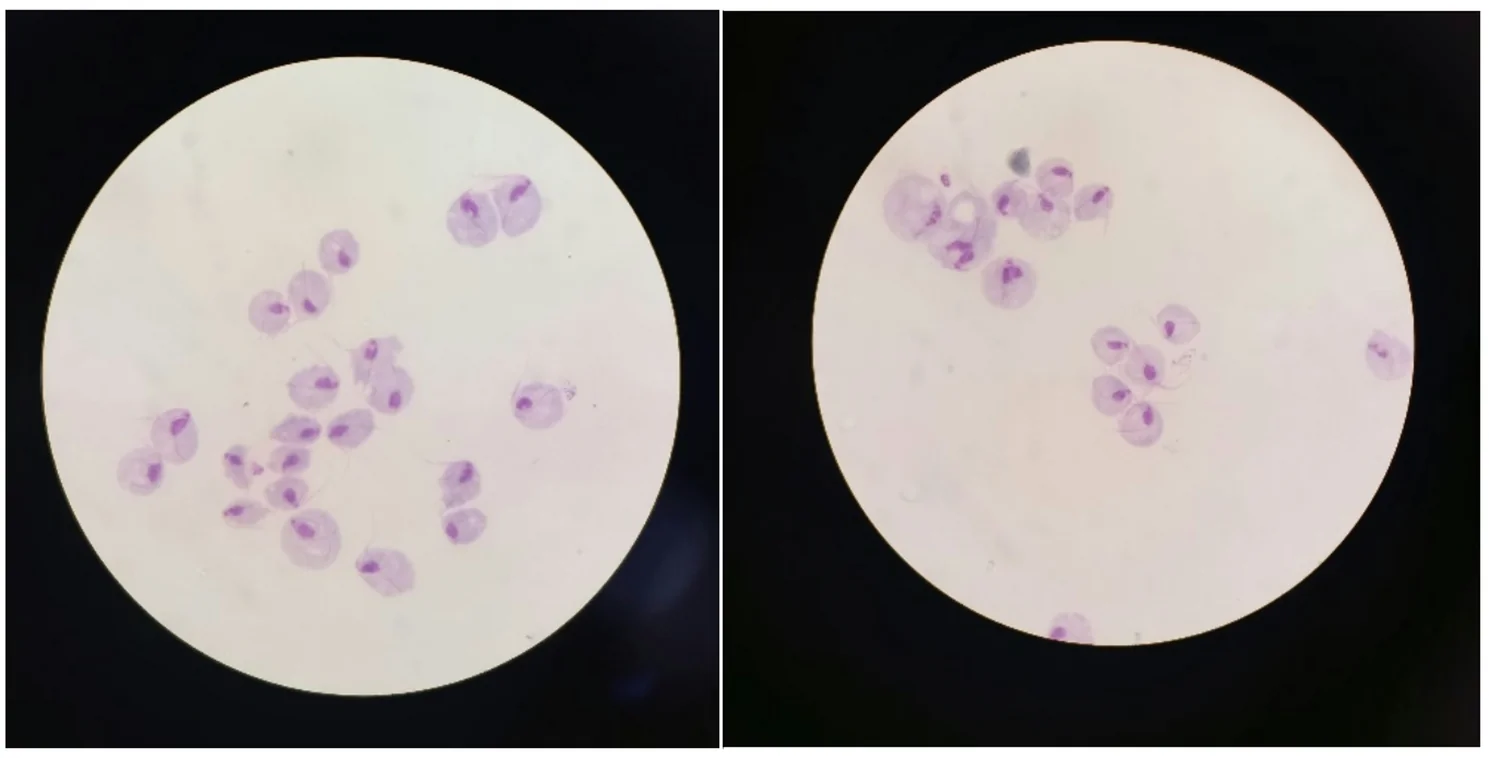
Morphology of *T. vaginalis* trophozoites from culture medium in a Giemsa-stained smear (100×)

On general examination, vital signs were stable and a regular fetal heart rate (FHR) of 145 bpm was recorded. Abdominal ultrasound confirmed a significant decrease in amniotic fluid (AFI = 5 cm) along with a live fetus in breech presentation. Laboratory diagnosis was pursued vigorously; urine samples were examined using Giemsa Staining, which confirmed the observation of motile and active *Trichomonas* trophozoites (Fig. 1). In addition to direct microscopic examination, samples were cultured in a specific *Trichomonas* culture medium, Trypticase-Yeast extract-Maltose (TYM), to increase diagnostic sensitivity, which was also positive (Video S1 and S2). In contrast, cultures for other common bacterial pathogens were negative. After confirming the rupture of the amniotic sac, necessary measures were taken, including prescribing a course of betamethasone for fetal lung maturation, neuroprotective magnesium sulfate, and GBS prophylaxis, and a standard dose of metronidazole (500 mg every 12 h for 7 days) was started for the patient to treat trichomoniasis.

Adherence to the prescribed course of metronidazole was primarily assessed via direct questioning of the patient during in-person follow-up consultations, where the patient reported excellent tolerability to the antimicrobial therapy, noting no complaints of nausea, vomiting, or other adverse effects. Furthermore, the tolerability of supportive care agents, including magnesium sulfate and betamethasone, was continuously monitored through serial physical examinations, which revealed no evidence of concerning side effects, confirming successful implementation and tolerance of the overall management plan. Short‑term follow‑up was performed during hospitalization with daily clinical and fetal monitoring; maternal symptoms resolved and laboratory testing confirmed eradication of infection prior to discharge. No longer‑term follow‑up data after discharge are available. The clinical timeline of presentation, diagnosis, treatment, and outcome is summarized in Table 2.

**Table 2:** Clinical timeline of presentation, diagnosis, and management

Stage	Description
Presentation	Presentation with premature preterm rupture of membranes (PPROM) at 25 weeks’ gestation
Diagnosis	Microbiological diagnosis confirmed at 25 + 1 weeks’ gestation
Treatment initiation	Initiation of targeted antimicrobial therapy and supportive management at 25 + 3 weeks’ gestation
Clinical course	Resolution of infection and improvement of clinical symptoms at 25 + 8 weeks’ gestation
Outcome	Discharge in stable condition at 25 + 10 weeks’ gestation

## Discussion

This case report highlights a clinically relevant association between sexually transmitted infections (STIs), such as trichomoniasis, and acute pregnancy complications, particularly preterm premature rupture of membranes (PROM). Trichomoniasis, often considered an infection limited to the vagina and urethra, has been suggested to be capable of involving higher tissue layers and may be associated with inflammatory responses [4].

The potential pathophysiological relationship between *T. vaginalis* infection and premature rupture of membranes may involve several biologically plausible mechanisms, particularly the exacerbation of local inflammatory processes. *Trichomonas* trophozoites have been suggested to contribute to alterations in the integrity of the amniotic membrane through the secretion of proteolytic enzymes, including cysteine proteinases, which are capable of degrading extracellular matrix components such as collagen and fibronectin that are essential for maintaining membrane strength. In parallel, *T. vaginalis* infection can induce inflammatory responses mediated by cytokines such as interleukin‑1 (IL‑1), interleukin‑6 (IL‑6), and tumor necrosis factor‑alpha (TNF‑α). These mediators may promote oxidative stress and activation of matrix metalloproteinases, further weakening the fetal membranes and increasing susceptibility to rupture [3, 5]. These processes may contribute to structural weakening of the membrane, which has been associated with PROM in late gestation (25 weeks), thereby increasing the risk of preterm birth and adverse fetal outcomes, including low birth weight (LBW). Physical examination findings (foamy discharge) and laboratory results from the Fern Test, Pooling Test, AmniSure Test, and Amniotic Fluid PAD Test supported the diagnosis in this case.

Several studies have shown an association between trichomoniasis infection and pregnancy complications such as preterm delivery, premature rupture of amniotic membranes, and low birth weight. In a recent case study by Sabet et al. (2025), a 39-year-old pregnant Australian woman with a history of chronic trichomoniasis was reported to have failed to respond to repeated metronidazole treatments. In this patient, uterine irritability and episodes of threatened preterm labor were observed repeatedly, a finding that emphasizes the possible role of inflammation caused by trichomoniasis infection in the occurrence of pregnancy complications including PROM. These observations suggest the need to review treatment protocols for resistant vaginal infections [8].

From an epidemiological perspective, Mabaso et al. (2020) demonstrated in a study of 362 pregnant African women that the prevalence of *T.vaginalis* infection detected by molecular methods was as high as 59.6%, while only 12.9% were identified by wet smear examination. Notably, all positive cases were asymptomatic, which highlights the potential importance of molecular screening during pregnancy, as asymptomatic infections have been associated with an elevated risk of premature rupture of membranes [9].

Similarly, Akinbo et al. (2017) reported a prevalence of trichomoniasis infection of 2.8% among pregnant women in Nigeria and identified a significant association between infection prevalence, gestational age, and women’s level of education. These findings suggest that sociodemographic factors may be associated with the occurrence or persistence of trichomoniasis infection and could be considered in the design of preventive strategies [10].

In a similar clinical report, Forbes et al. (2015) described a case of *T. vaginalis* infection in a 28‑year‑old pregnant British woman who presented with prominent symptoms, including foul‑smelling discharge, pain during intercourse, and post‑coital bleeding. In this patient, other tests for genital infections, including *Chlamydia trachomatis* and *Neisseria gonorrhoeae*, were negative, and *T. vaginalis* was identified as the only detected pathogen. This observation underscores that, even in the absence of other common pathogens, trichomoniasis may be associated with pronounced inflammatory responses, which could potentially contribute to the weakening of amniotic membranes and an increased susceptibility to premature rupture [11].

In the present report, similar findings were observed regarding the association between vaginal infection and amniotic membrane rupture. Although the definitive cause of PROM is multifactorial, our findings suggest a possible association between the presence of pathogens such as *T.vaginalis* and alterations in the integrity of the amniotic membranes, potentially mediated by inflammatory processes, changes in vaginal pH, and tissue damage. Accordingly, early identification and management of vaginal infections during pregnancy may be beneficial and warrant consideration as part of strategies aimed at reducing adverse pregnancy outcomes. Given the observational nature of the study, causal relationships cannot be inferred, and further longitudinal and interventional studies are required to confirm these associations.

The strengths of this report include the clear description of the observed association between *T. vaginalis* infection and premature rupture of membranes (PROM) in a pregnant woman, which underscores the potential importance of diagnosing and monitoring this infection during pregnancy. The presentation of detailed clinical and laboratory findings allows for careful confirmation of the diagnosis and assessment of related complications, making this report a valuable clinical observation for obstetric practitioners.

Furthermore, the emphasis on the need for screening and early diagnosis of trichomoniasis in pregnant women suggests that timely management may help to lower the likelihood of adverse outcomes such as PROM and preterm delivery. In addition, raising awareness among healthcare staff about the possible complications associated with this infection may contribute to better maternal and neonatal outcomes.

Several limitations of this report should be acknowledged. First, the available information regarding the patient’s medical history and other potential risk factors was incomplete. In addition, limited documentation of certain behavioral and socioeconomic parameters restricts the ability to fully exclude residual confounding factors or interactions with other contributors that may have influenced the observed outcome.

Second, although the scientific rationale of this report is based on the observed association between *T. vaginalis* infection and premature rupture of membranes (PROM), this observation must be interpreted cautiously. Common predisposing factors for PROM, such as previous preterm birth or multiple pregnancy, were absent, and the infection was laboratory confirmed; therefore, it is biologically plausible that chronic inflammation related to *T. vaginalis* infection may have contributed to alterations in the structural integrity of the fetal membranes. Nevertheless, as this is an observational single‑case report, a causal relationship cannot be established, and this association remains speculative.

Third, objective assessment of inflammation could have strengthened the biological interpretation of this case. However, inflammatory parameters such as white blood cell count, erythrocyte sedimentation rate, and cytokine profiles were not measured. This omission represents one of the most important limitations of the present report. Future investigations should incorporate systematic evaluation of inflammatory markers and, where feasible, assessment for bacterial vaginosis to better elucidate underlying pathophysiological mechanisms.

Fourth, urine Giemsa staining was used as an auxiliary diagnostic method and is not considered a standard diagnostic approach for *T. vaginalis*. Although this limitation was mitigated by confirmatory culture on *Trichomonas*‑specific medium (TYM), which established the definitive diagnosis, reliance on non‑standard auxiliary testing remains a methodological constraint.

Fifth, a significant practical limitation was the non‑adherence of the patient’s sexual partner to simultaneous evaluation and treatment. Despite thorough counseling regarding the importance of partner management to prevent reinfection and subsequent complications, follow‑up was unsuccessful due to the partner’s non‑cooperation. This highlights real‑world challenges in the clinical management of sexually transmitted infections during pregnancy, where social and behavioral factors may undermine the effectiveness of standard treatment protocols.

Finally, the single‑case nature of this report limits the generalizability of the findings. Interventional studies and prospective cohort investigations with larger sample sizes are required to more robustly examine the association between trichomoniasis and premature rupture of the amniotic membranes. Additionally, future studies assessing the impact of timely treatment on PROM risk, as well as genetic and molecular investigations aimed at identifying susceptibility factors, may further clarify the biological mechanisms involved and support the development of targeted preventive and therapeutic strategies.

## Conclusion

This case illustrates a possible association between vaginal and urinary *T. vaginalis* infection and premature rupture of membranes (PROM) in the second trimester of pregnancy. Prompt diagnosis using a combination of clinical evaluation and laboratory confirmation—including Fern, Pooling, AmniSure, and PAD tests, as well as microscopic and culture-based identification—enabled timely initiation of targeted therapy.

The report highlights the importance of routine screening for sexually transmitted infections in pregnant women, even in the presence of atypical symptoms such as urinary incontinence, given their potential link with serious obstetric complications like PROM and preterm labor. Close monitoring and appropriate management after confirmed diagnosis may help mitigate adverse outcomes; nevertheless, as this is an isolated case, a direct causal relationship cannot be established.

Overall, these findings support the value of heightened clinical awareness and adherence to evidence-based guidelines for the diagnosis and management of trichomoniasis during pregnancy. Further prospective and interventional research is necessary to clarify the role of such infections in the pathogenesis and prevention of acute obstetric complications.

## Supplementary Information

Supplementary Material 1. Videos S1 and S2: Motility patterns of *Trichomonas vaginalis* trophozoites. Giemsa-stained smear preparation of urine sediment, 100x magnification.

## Data Availability

The datasets generated and/or analysed during the current study are available from the corresponding author on reasonable request.
